# Review of the Flight Control Method of a Bird-like Flapping-Wing Air Vehicle

**DOI:** 10.3390/mi14081547

**Published:** 2023-07-31

**Authors:** Xiaoqing Fang, Yian Wen, Zhida Gao, Kai Gao, Qi Luo, Hui Peng, Ronghua Du

**Affiliations:** 1 College of Automotive and Mechanical Engineering, Changsha University of Science & Technology, Changsha 410114, China; xiaoqingfang089@gmail.com (X.F.); gaodamu828@163.com (Z.G.); luoqi@csust.edu.cn (Q.L.); csdrh@csust.edu.cn (R.D.); 2 College of Electrical and Information Engineering, Changsha University of Science & Technology, Changsha 410114, China; 202103130815@stu.csust.edu; 3 Hunan Key Laboratory of Smart Roadway and Cooperative Vehicle-Infrastructure Systems, Changsha 410114, China; 4 School of Computer Science and Engineering, Central South University, Changsha 410075, China; hui-peng@csu.edu.cn

**Keywords:** flight control, position control, trajectory tracking control, formation control

## Abstract

The Bird-like Flapping-wing Air Vehicle (BFAV) is a robotic innovation that emulates the flight patterns of birds. In comparison to fixed-wing and rotary-wing air vehicles, the BFAV offers superior attributes such as stealth, enhanced maneuverability, strong adaptability, and low noise, which render the BFAV a promising prospect for numerous applications. Consequently, it represents a crucial direction of research in the field of air vehicles for the foreseeable future. However, the flapping-wing vehicle is a nonlinear and unsteady system, posing significant challenges for BFAV to achieve autonomous flying since it is difficult to analyze and characterize using traditional methods and aerodynamics. Hence, flight control as a major key for flapping-wing air vehicles to achieve autonomous flight garners considerable attention from scholars. This paper presents an exposition of the flight principles of BFAV, followed by a comprehensive analysis of various significant factors that impact bird flight. Subsequently, a review of the existing literature on flight control in BFAV is conducted, and the flight control of BFAV is categorized into three distinct components: position control, trajectory tracking control, and formation control. Additionally, the latest advancements in control algorithms for each component are deliberated and analyzed. Ultimately, a projection on forthcoming directions of research is presented.

## 1. Introduction

Researchers have drawn inspiration from flying animals to create various flight systems, including fixed-wing, rotary-wing, and flapping-wing systems [1,2]. Therein, fixed-wing systems lack flexibility and are incapable of hovering [3]. However, the efficiency of rotor wing systems in generating lift during hovering flight is reduced at low Reynolds numbers [4]. Flapping-wing vehicles, developed based on bionic principles [5,6], offer advantages over fixed and rotary wings, including high energy utilization, low noise [7], strong maneuverability, and stealth [8,9]. And flapping-wing vehicles possess the capability to scrutinize the underlying mechanisms of agile flight in actual avian and insect species [10], as well as accomplish intricate and challenging objectives via clustering behaviors [11]. Consequently, flapping-wing air vehicles exhibit auspicious prospects and substantial potential for employment in many domains. The civilian side can be utilized for environmental assessment, remote sensing, security surveillance, border patrol, etc., as well as for reconnaissance missions in physically difficult-to-reach and hazardous locations [12] to carry out risky or dirty tasks like radiation detection, chemical spill cleanup, or working in areas with high electromagnetic intensity. The considerable dimensions of big BFAVs, in contrast to flapping-wing air vehicles resembling insects, coupled with their large load capacity, render them suitable for specialized missions. For instance, BFAVs outfitted with miniature cameras and high-energy microbombs can function as covert and precise weapons for targeted attacks [13].

**Figure 1:**
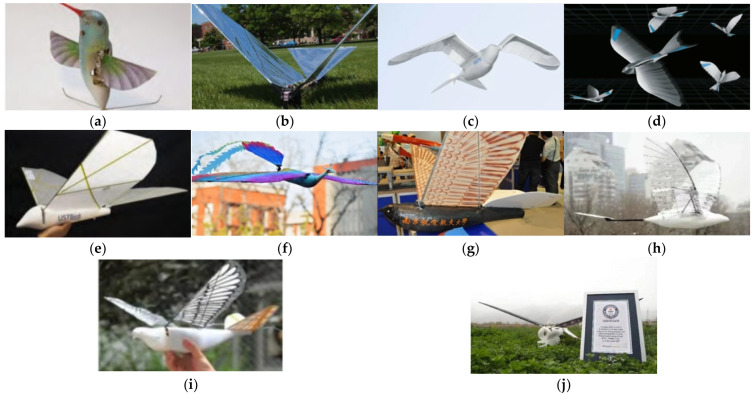
International BFAVs: (**a**) Nano Hummingbird; (**b**) Robo Raven; (**c**) SmartBird; (**d**) BionicSwifts; (**e**) USTBird; (**f**) HIT-Phoenix; (**g**) Sky Hawk; (**h**) Two-jointed bird; (**i**) Dove; (**j**) Cloudy Owl. These figures are taken from the internet.

Flapping-wing air vehicles have become a research hotspot [14,15] in recent years due to the development of microelectronics, intelligent materials, precision machining, and other high technologies, as well as the demand for progress in industrial technology and education. This bionic technology has advanced significantly. Internationally, some technology corporations and universities have conducted in-depth research on flapping-wing air vehicles, producing physical prototypes that highly mimic birds in nature in form and function and have successfully achieved autonomous flight. The main ones are Nano Hummingbird [16] (Figure 1a) from the American AeroVironment Corporation, Robo Raven [17] (Figure 1b) from the University of Maryland, the bionic silver gull “SmartBird” (Figure 1c) and the bionic rainbird “BionicSwift” (Figure 1d) from the German Festo, “USTBird” by He Wei’s team at the University of Science and Technology Beijing [18,19,20,21] (Figure 1e), the bionic phoenix “HIT-Hawk” and “HIT-Phoenix” (Figure 1f) [22] by Xu Wenfu’s team at Harbin Institute of Technology (Shenzhen), “Sky Hawk” (Figure 1g) by Ang Haisong’s team at the Nanjing University of Aeronautics and Astronautics, “Big Two-jointed Bird” (Figure 1h) by the team of Beihang University, and the “Dove” [23] (Figure 1i) and “Cloudy Owl” [24] (Figure 1j) by the team of Song Bifeng from Northwestern Polytechnical University.

Some of the most representative studies are the bionic rainbird “BionicSwift” from the German Festo and “Cloudy Owl” from Northwestern Polytechnical University. Festo’s bionic swift, “BionicSwift”, has a body length of 44.5 cm, a wingspan of 68 cm, and a weight of only 42 g. The bionic air vehicle is quick and flexible and can make tight maneuvers and flying loops. Additionally, actual flight tests have shown that five BionicSwifts may maneuver in unison and autonomously in the designated airspace while engaging with the radio-based indoor GPS. To push the boundaries of endurance, load capacity, and environmental adaptability, Prof. Song Bifeng’s team has been designing more bionic and effective flapping-wing air vehicles. The team’s “Cloudy Owl” flapping-wing air vehicle achieved a new world record in 2022 by successfully flying an autonomous course for 154 min and making a soft landing.

Flight control, as an integral part of the research on flapping-wing air vehicles, is the basis for the realization of autonomous flight, trajectory orchestration, and other capabilities of the air vehicle. A highly bird-like flapping-wing air vehicle not only needs to mimic the shape and flight mode of a bird but also needs to be more capable of accomplishing complex flight maneuvers such as turning, diving, and flipping like a bird. In consideration of the fact that the flapping-wing air vehicle is a nonlinear and unsteady system, it is susceptible to factors such as environmental disturbances. Moreover, the mass and size of the flapping-wing system greatly limit energy, sensors, and airborne processing, leading to a sharp decline in performance. Hence, it is imperative to use well-defined flight control algorithms to improve the stability of the system. The key way to obtain fine vehicle control and make the flapping-wing air vehicle fly more nimbly, steadily, and safely is through effective and precise algorithms. Therefore, excellent flight control algorithms are crucial to flapping-wing air vehicles’ flight performance and application prospects. Based on this background, this paper investigates the flight control algorithm of BFAV with nonlinearity, parameter coupling, and uncertainty and summarizes the research progress of the BFAV flight control algorithm so far.

**Figure 2:**
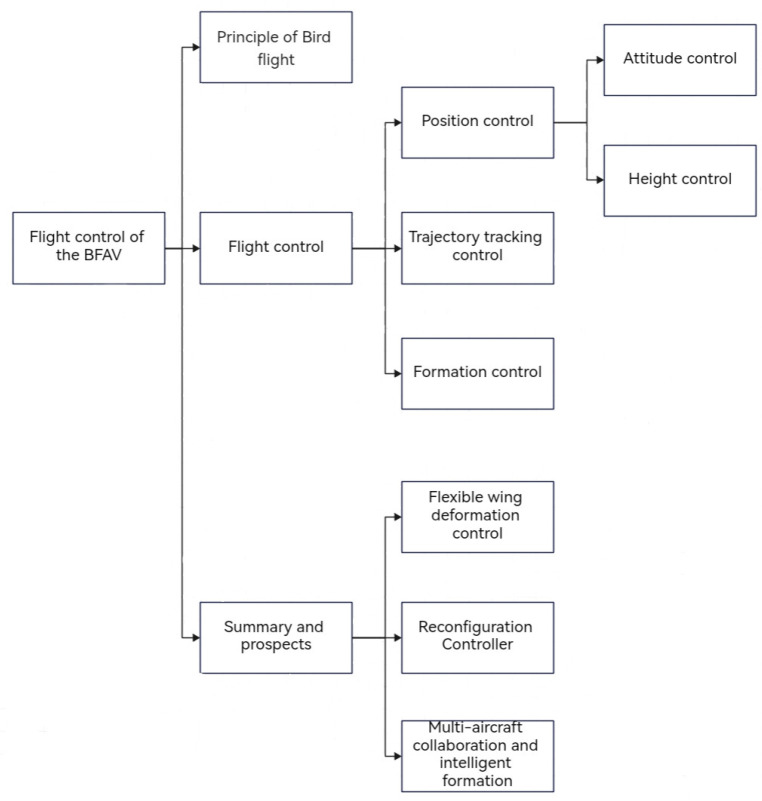
Flight Control system of the BFAV.

The structure of this essay is as follows (Figure 2). The first part introduces the principles of bird flight. The second part demonstrates the latest development of the BFAV’s flight control algorithm from three perspectives: position control (including attitude control and height control), trajectory tracking control, and formation control, and the third part summarizes the progress of the BFAV and offers a prediction for the future of its research.

## 2. Mechanism of Bird Flight

The remarkable flying capabilities of insects and birds in their natural habitats have been extensively studied [25]. In an effort to create a vehicle with similar flight abilities and effective endurance, researchers have endeavored to replicate the flight principles of birds. 

**Figure 3:**
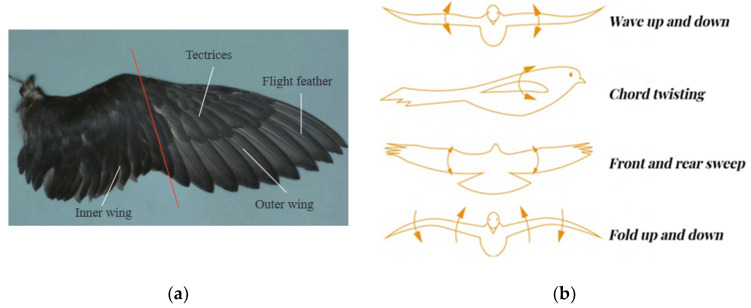
Structure and Schematic Diagram of Bird Wing: (**a**) Structure of Bird Wing. Reprinted with permission from Ref. [26]. 2022, Chinese Journal of Engineering; (**b**) Schematic diagram of bird wings waving, twisting, sweeping, and folding. Reprinted with permission from Ref. [29]. 2022, SCIENTIA SINICA Technologica.

The wing, which is the most vital component of avian flight, is a complex structure consisting of primary and secondary flight feathers, covering feathers, and down feathers that span the entire wing. This intricate structure can be divided into two distinct parts, namely the inner and outer wings [26], as depicted in Figure 3a. When flying, birds periodically flutter their wings in a certain rhythm to provide the lift and thrust needed for flight [27]. A typical macroscopic flutter of a bird’s wings consists of waving, twisting, sweeping, and folding [28], as shown in Figure 3b. Among them, waving motion is the most fundamental type of flapping, and other motions play distinct roles by superimposing waving [29]. And for the BFAV, it is mainly based on the Anti-Karman vortex street principle to obtain propulsion in order to achieve effective endurance flight performance and maximum propulsion efficiency [27]. The wake vortex generated by fluttering the wing forms the Anti-Karman vortex street, which causes the flow field in the wake of the wing to form thrust. At a particular forward flight speed, the wing shape will be similar to that of a fixed wing so as to create enough quasi-constant aerodynamic lift.

The wing is an integral part of a flapping-wing air vehicle. The different forms of wing bending during flapping make the wings flexible and contribute significantly to the aerodynamic efficiency of flapping motion [11]. Furthermore, additional research has demonstrated that the folding action of the wings can improve their efficiency to produce lift and reduce energy loss [30]. Under inertial and aerodynamic loads, different flutter area distributions generate varying degrees of torsional deformation in their various spreading profiles. This twist modifies the magnitude of flapping lift by altering the size of the leading-edge vortex and the direction of the differential pressure forces on the upper and lower surfaces of the wing [29]. To achieve wing deformation and subsequently control the forces interacting with the air, the BFAV uses a flexible wing.

The aerial flight environment is complex and variable nonetheless, as it incorporates unstable flows from a variety of origins, such as wind gusts and turbulence that may be suffered during flight [31]. When confronted with wind gusts, birds are enabled to rapidly adjust their wing heights and rotate around their shoulders to make their wings rise with the gusts in order to minimize the impact of the gusts. This preflex mechanism suppresses the gust pulse through inertial effects and reduces the movement of the bird’s torso and head [32]. Atmospheric flows are usually turbulent. Thus, stabilizing turbulence is critical for flying animals. In the face of turbulence, birds have complex coping strategies. This includes making both active and passive adjustments to the kinematics of wing beats and also adapting by adjusting the shape and angle of the wings. Additionally, birds are able to sense and utilize the approximately stationary flow structure in turbulence to reduce the influence of turbulence and improve flight efficiency. By investigating the issue of how feathers stick together to form a deformed wing, Matloff et al. discovered that when a bird moves its skeleton to alter the shape of the wing plane, the flight feathers on the wings are redistributed as a way to be robust to turbulence [33]. Designing a hinged wing by using the shoulder joint as a hinged suspension system can be applied to small-scale vehicles with proper adaptations. Implementing a similar preflex mechanism in a flapping-wing air vehicle can also help mitigate the effects of gusts and turbulence without increasing the computational burden [32]. In addition, the tail of birds also plays an important role in their flight. Through the coordination of their wings and tails, birds perform various maneuvers of aerial flight, such as spins, dives, and flips. Most studies of the aerodynamic properties of an air vehicle’s wing and tail are currently conducted using CFD simulations [34,35,36]. It was found that the overall efficiency and average thrust of each wing increased by 17% and 126%, respectively, when compared to a single flapping-wing air vehicle with proper tail position and a setting angle [35]. A suitable tail section can extend the vehicle’s range in addition to enhancing flight maneuverability, which is advantageous for missions involving long flight durations. In the flight control of BFAVs, the configuration of the tail section of the flapping-wing air vehicle can provide many benefits, including static stability and easier control methods [36]. Moreover, the tailed vehicle’s tail is essential for maintaining its attitude [37]. Flight maneuvers such as roll, and pitch are possible due to the exact deflection of the tail control surface utilized by the controller in conjunction with wing flapping. The synergy between the wing and the tail accomplishes the agility of the BFAV.

In conclusion, the geometry of avian wings and their deformation, the arrangement of feathers, and the synergistic cooperation between wings and tail are critical determinants of avian flight. The predominant mechanism of flight control is achieved through the flapping of wings and tail in unison, as no appropriate material has been identified for the distributed control of the wings of a flapping-wing air vehicle, precluding the autonomous arrangement of feathers akin to that of birds. Despite the potential benefits of this mechanism, its practical application is still fraught with numerous challenges. One such challenge is the intricate interplay between the non-constant wing wake flow and the wake, which often eludes explicit modeling [36]. This complex wake aerodynamics can significantly affect the stability of the flapping-wing air vehicle. Consequently, researchers have devoted considerable effort to investigating flight control algorithms, as reliable flight control is a crucial determinant of mission success. The study of flight control algorithms not only helps to improve the flight performance of BFAVs but can also contribute to the better development of the field. In the next section, we will highlight the flight control algorithms of recent years.

## 3. Flight Control

The control algorithm of BFAV has been a popular subject of study in the academic realm. The flight control algorithm can be categorized into three primary components: position control, trajectory tracking control, and formation control. Position control is the fundamental element of the control system, encompassing attitude and height control, which guarantee the air vehicle’s maintenance of the correct attitude and height during flight. Trajectory tracking control directs the air vehicle to follow a predetermined trajectory and can be utilized for various tasks such as cruise and search. Eventually, the implementation of formation control will allow for collaborative flight among multiple BFAVs, thereby enabling the execution of mission assignments, cluster searches, and other related activities. 

### 3.1. Position Control

#### 3.1.1. Attitude Control

**Figure 4:**
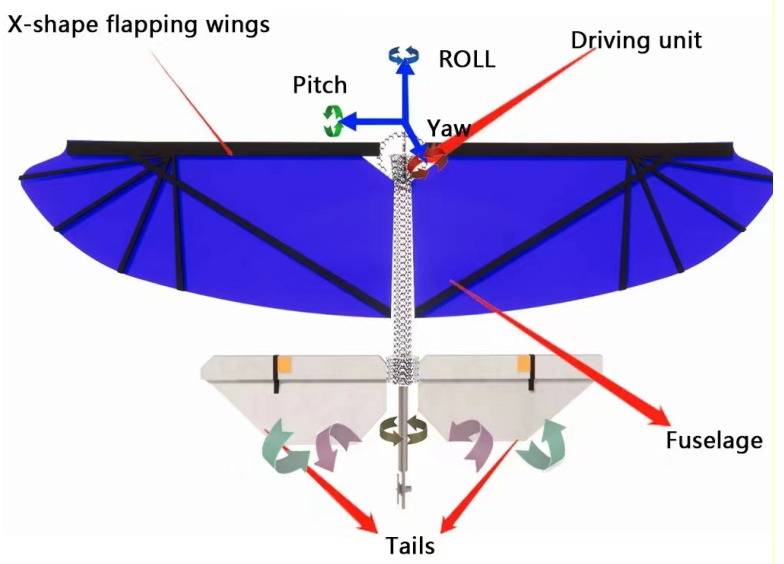
Conceptual view of Beihawk. Reproduced with permission from ref. [37]. 2021, Elsevier Masson SAS.

Attitude control is essential for achieving precise orientation and tracking flight for all types of air vehicles [38]. The adjustment of pitch, yaw, and roll attitudes is an integral aspect of attitude control. With the goal of achieving the flapping wing microair vehicle’s (FWMAV) pitch control, Wang et al. have proposed a dynamic linearization approach based on a model-free adaptive control scheme [39]. This approach incorporates an anti-saturation compensator to prevent input saturation, thereby enhancing the efficacy of the model-free adaptive control scheme for pitch control in comparison to conventional methods. Furthermore, Wang et al. have implemented the bionic wing-tail contact mechanism for pitch control through a comprehensive investigation of the biological excitation mechanism. They have established a precise tail control model for the air vehicle and designed a frequency-dependent tail controller [40] that can effectively achieve pitch stability by utilizing the flapping-induced flow. Birds rely on this mechanism for attitude control. Fully referencing it in a flapping-wing air vehicle facilitates the design of the controller and further enhances the bionic level of the flapping-wing air vehicle. Jiao et al. [37] incorporated an X-shaped flap downstream of the main flap for the roll, pitch, and yaw control surfaces on the tail, as well as the tail rotor, as illustrated in Figure 4. The “Northern Hawk” unsteady aerodynamic model was utilized, which accounts for unsteady effects and wing-tail interactions. The pitch torque is produced by the coordinated motion of the two tail control surfaces; the roll torque is generated by the different polarities of the deflection; and a bi-directional controllable tail rotor assembly is installed to actively generate the yaw torque.

The bionic flapping-wing vehicle is widely recognized as a nonlinear and unsteady system [41], characterized by nonlinear, time-varying, and highly coupled features that pose significant challenges for attitude control. Prior research has mentioned conventional attitude control issues for stiff bodies in three dimensions, as documented in [42,43], which highlight structural vibrations and external disturbances as typical problems arising from the use of flexible components. Despite the numerous control schemes proposed by researchers to mitigate these issues, each method has its limitations. Consequently, contemporary attitude control predominantly employs a hybrid control strategy, which leverages the advantages and limitations of individual control techniques to supplement each other. This approach is exemplified by sliding mode control (SMC). Bluman et al. demonstrated that SMC effectively mitigates the uncertainty of the flapping-wing air vehicle’s model and external disturbances that microair vehicles may encounter [44]. Due to the requirement of knowledge pertaining to the matching requirements for the system’s uncertainty terms and logical switching utilizing switching functions to maintain the system in sliding mode, jitter remains an inevitable consequence of SMC. Hence, Hang Li et al. have proposed the integration of adaptive control [38] with SMC to address its inherent limitations. Adaptive control is utilized to address the limitations of SMC, while SMC exhibits notable resilience to sinusoidal disturbance airflow and promotes the stability of flapping-wing air vehicle attitude control. Additionally, SMC compensates for the inadequate responsiveness of adaptive control in the presence of rapidly changing parameters. Furthermore, Qian et al. introduced a novel hybrid attitude control approach based on quaternion, which comprehensively accounts for the underdriven and induced aerodynamic effects in the development of a flapping-wing flight controller, marking a significant advancement in this field [45]. The successful execution of stable hovering and forward flight by the flapping-wing air vehicle has provided a theoretical foundation for addressing the issue of three-dimensional attitude control with underactuation in the yaw axis.

#### 3.1.2. Height Control

The stable and precise control of flight altitude is a crucial aspect of flapping-wing air vehicle flight control, as acknowledged in the literature [46]. Accurate altitude adjustment significantly impacts flight performance. FWMAV has a limited carrying capacity due to its small mass and size and has an urgent demand for sophisticated and miniaturized sensors. In the past, research on flapping-wing air vehicles primarily concentrated on control algorithms, owing to the constraints of scientific and technological advancements. Subsequently, with the rapid development of science and technology, researchers began to greatly improve the flapping-wing structure and hardware system. Verboom et al. introduced the initial dependable onboard state estimation and onboard control for the flapping wing microair vehicle (FWMAV) by utilizing a barometer to determine atmospheric pressure [47]. A PI height controller was designed based on a fixed reference pressure set in advance, which enabled the vehicle to achieve successful hovering, albeit with a large hovering range and low performance. Ryu and Kim et al. [48] employed the barometer for height control and developed an FWMAV with dual main flaps and tail fins, utilizing a PID controller with an optimized structure. The vehicle was able to sustain a near-optimal altitude for an extended duration with minimal deviation owing to this design. However, the imprecise nature of barometric measurements and their susceptibility to atmospheric conditions pose significant limitations. Conversely, cameras do not accumulate errors, exhibit robustness against external disturbances, and have versatile applicability. As a result, vision systems have been leveraged to assist height regulation by controllers [46,49,50,51]. For instance, He et al. introduced a model-based controller that utilizes an external vision system as the height sensor and employs a PID control algorithm based on vision measurements to facilitate the flapping-wing air vehicle’s height-holding control [51]. This approach exhibits minimal errors and enables the flapping-wing vehicle to precisely track the desired altitude.

**Figure 5:**
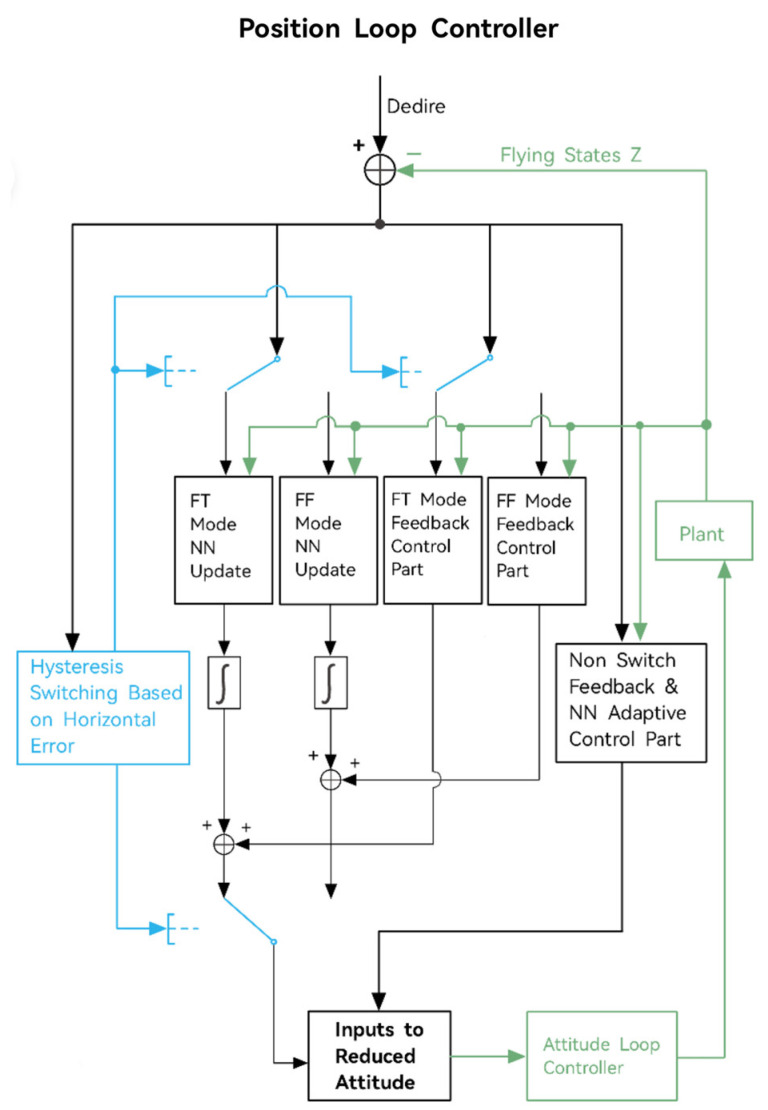
Schematic for the system block diagram and the control task. Reproduced with permission from ref. [54]. 2022, IEEE.

The PID control technique is a conventional method for controlling the height of flapping-wing air vehicles in flight control studies, but its effectiveness is restricted when applied to complex nonlinear systems and complex signal tracking. Consequently, researchers have shifted their focus towards utilizing more advanced control algorithms, such as the adaptable neural network algorithm. In this regard, AI-Mahasneh et al. developed an adaptive control system that employs a generalized regression neural network (GRNN) as the height controller for FWMAVs [52]. Upon comparison with a conventional PID controller, it was discovered that the control performance of the PID deteriorates swiftly with alterations in the input, whereas the GRNN controller can readily adjust to input changes. Furthermore, the performance of the GRNN-based controller enhances over time as the neural network parameters approach the optimal parameters. This substantiates the superiority of the GRNN controller over the classic PID controller. Additionally, Mou et al. conducted a study on the challenges associated with height control in the presence of disturbances [53]. They proposed an active perturbation suppression controller to estimate and suppress both internal and external disturbances, which effectively mitigates overshoot and enhances height control accuracy. In the same year, Qian et al. introduced a novel hybrid neural network-based switching control strategy [54], incorporating a lateral position error switching control strategy and an update strategy for the position controller. Figure 5 displays the system’s block diagram and control task, with “FF mode” denoting the forward flying mode, “FT mode” representing the fine-tuning mode, and “NN” signifying the neural network. The experimental outcomes demonstrate that the proposed strategy effectively stabilizes the flapping-wing air vehicle at the intended 3D location with high dependability and efficacy.

### 3.2. Trajectory Tracking Control

The utilization of trajectory tracking control in pre-planning and optimizing the flight trajectory of BFAVs based on attitude and height control can enhance the stability and accuracy of the air vehicle during flight. Consequently, trajectory tracking control assumes a crucial role in the operation of flapping-wing air vehicles.

The control algorithm for trajectory tracking in a flapping-wing air vehicle necessitates consideration of various factors involved in the flight itself, such as nonlinear modeling, time-varying disturbances, and unidentified external uncertainties, which can potentially impact the stability and precision of the control system. Research has shown that neural network techniques are proficient in managing nonlinear systems with exceptional approximation [55,56]. He et al. developed a neural network controller with full-state feedback and output feedback to mitigate the uncertainties associated with nonlinear FWMAV [57]. The controller demonstrated effective trajectory tracking. The researchers conducted a simulation to compare the performance of four controllers: the PID controller, the model-based controller, the output feedback neural network controller, and the full-state feedback neural network controller. The comparative analysis reveals that the PID controller exhibits considerable tracking error and inadequate performance in tracking the desired trajectory, thereby rendering it unsuitable as a standalone controller for trajectory tracking. The model-based controller, on the other hand, can accomplish trajectory tracking, but it necessitates the acquisition of all system parameters. A comprehensive evaluation indicates that full-state feedback neural network control yields the optimal tracking effect. Nevertheless, the simulation outcomes for the diverse controllers do not accurately reflect the optimal trajectory tracking outcome for this type of controller. For instance, Wissa et al. have demonstrated the effectiveness of PID control through the design of an integrated two degree-of-freedom controller [58], which enables robust trajectory tracking even in the presence of external perturbations. Furthermore, they have developed a high-fidelity nonlinear and time-periodic six degrees of freedom dynamics model of the FWMAV for model-based control purposes, utilizing averaging techniques to propose a novel Lyapunov-based closed-loop control approach [59]. Upon achieving dependable trajectory tracking through the use of feasible control inputs, the controller exhibited resilient performance in the face of external perturbations and parameter uncertainties. In light of this high-fidelity model, a novel integral-command filter block backstepping controller was developed by researchers [60]. This controller surpasses Lyapunov-based closed-loop control methods in terms of its adaptability and performance when confronted with constant and time-varying matching and mismatching disturbances. It can effectively track any desired reference trajectory with practical control.

Model-based controllers [61] enable the simulation and analysis of the model, thereby enhancing control accuracy. However, their implementation requires the development of intricate models beforehand and a high level of modeling proficiency. Conversely, model-free controllers do not require pre-modeling and are more flexible in unfamiliar environments. Table 1 outlines the model-free control techniques typically employed in trajectory tracking control, along with their advantages and disadvantages.

Furthermore, practical applications of FWMAVs are often impeded by challenges such as input deadband [62] and input restrictions. To address the issue of input dead zones, Tang et al. developed a dynamic surface controller based on a predefined performance function and an adaptive neural network estimator [63]. This approach effectively mitigates the tracking inaccuracy associated with dead zones and facilitates the control of position and attitude trajectory tracking for flapping-wing air vehicles. The adaptive tracking controller for the FWMAV system designed by Qian and Fang et al. ensures satisfactory control performance even in the presence of various perturbations and input constraints [64].

**Table 1:** Advantages and disadvantages of common model-free control methods for trajectory tracking.

Control Method	Advantage	Disadvantage
PID Control [57,65]	Simple and easy to implement, fast response time, and a wide range of applications	Dependent on precise adjustment of parameters, sensitive to system modeling errors, and not adaptive
Adaptive Control [18,66]	Self-adjustable control parameters according to the real-time status of the system	Adaptive factors are process independent and require additional conditions
Neural Network Control [67]	Strong nonlinear approximation capability is applicable to most nonlinear control problems and can effectively deal with systems with incorrect mathematical descriptions	A large amount of training data is required, which is computationally intensive and requires high hardware requirements, and fewer samples will lead to poor system performance
Iterative Learning Control [68]	No need for system modeling and parameter estimation	Requires large amounts of experimental data and computational resources, sometimes with slow convergence and overfitting
Fuzzy Control [69]	No accurate physical model is required, it is easy to design and apply, and the adjustment of parameters can effectively handle nonlinearities	For inexperienced people, it is harder to choose the right parameters

### 3.3. Formation Control

The completion of intricate missions often requires the use of multiple flapping-wing air vehicles, as relying on a single vehicle can prove challenging. The advancement of group intelligence has led to increased interest in the cluster formation technology of these vehicles. Formation flying is a flight strategy that maximizes the utilization of airflow and aerodynamic effects through the formation of multiple vehicles in flight with predetermined positional alignments and attitudes. For instance, a rear vehicle may utilize vortices generated by a front vehicle in the formation to reduce its own resistance and energy consumption. Furthermore, interactions among the vehicles have the potential to significantly decrease air resistance and enhance overall flight efficacy. Therefore, flapping-wing air vehicles’ cluster formation transformation, by emulating the characteristics and laws of bird clusters, can achieve optimal and balanced energy consumption and enhance long-distance endurance, as evidenced by previous research [70].

Significant avian species undergo extensive migration with the changing of the seasons. Wild geese, for example, migrate in formations that take the shape of “V,” “I,” or “L.” Among these formations, the “V” formation is deemed the most effective for achieving group energy savings, a phenomenon known as the “Wild Geese Queue Effect” [71,72]. Numerical simulations conducted by Wee-Beng Tay et al. [73] further support the benefits of V-formations in improving the range of flapping-wing systems. As a result, the majority of studies on BFAV formation flight control focus on the “V” formation. Andersson et al. [74] conducted a thorough investigation into the “V” formation of birds and determined that the acute angle “V” formation is optimal for larger birds, with energy savings being primarily realized by the followers. Conversely, the obtuse angle “V” formation is more suitable for smaller birds, with energy savings being relatively consistent across all positions.

An optimal flight arrangement has the potential to mitigate the energy consumption of the collective and enhance its range. Nevertheless, the alterations in airflow during the flight are multifaceted, and the crux of formation flight lies in the ability to promptly adapt to environmental changes. The realization of collaborative control of multiple air vehicles is the prerequisite guarantee for the completion of excellent flight arrangements and the realization of formation flight. In contrast to the flight control of a solitary flapping-wing air vehicle, the management of formation flight necessitates not only the appropriate control of each air vehicle but also the synchronized administration of multiple air vehicles to avert collisions during formation alterations. The primary techniques for formation control comprise the leader-follower method, the behavior-based method, the virtual structure method, and the consistency method. The advantages and disadvantages of each of the four control approaches are enumerated in Table 2 of this composition. The leader-follower approach is primarily characterized by the leader’s adherence to a predetermined trajectory, while the follower maintains a relative position by tracking the leader’s speed, yaw angle, and altitude [75]. This approach has gained widespread adoption in formation control due to its simple control structure and scalability [76]. Yuanpeng Wang has developed a bionic flapping-wing formation flight control method based on the leader-follower approach to attain straight-line flight, circular trajectory flight, and formation transformation within a triangular formation. The method incorporates the formation’s uniformity in terms of speed, heading, and altitude. The approach employs behavior-based simulation to emulate biological response behavior by defining fundamental control behaviors of the air vehicle and integrating them to accomplish sophisticated behaviors of the formation, such as following and cooperation [77]. The virtual structure approach posits that the formation is a virtual rigid body, wherein each member is a fixed point within the said structure, and the formation is sustained by maintaining a specific distance between each member and its corresponding fixed point within the virtual rigid body [78]. Conversely, the consistency technique governs the relative velocity and relative position of the formation’s members to ensure uniformity in their motion and behavior. It has become a relatively active research topic in this field due to its high reliability, good self-healing, and scalability.

**Figure 6:**
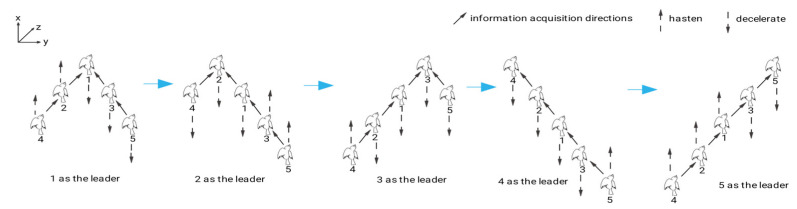
Reconfiguration of flapping-wing air vehicles. Reprinted/adapted with permission from Ref. [83]. 2021, ACTA AUTOMATICA SINICA.

Furthermore, communication and obstacle circumvention have emerged as primary concerns in the realm of formation control. The existing literature has identified artificial potential fields [79] and distributed model predictive control [80] as the predominant approaches employed to address the obstacle avoidance issue. Wu et al. have effectively addressed the obstacle avoidance issue by integrating the enhanced consistency algorithm and particle swarm algorithm to circumvent static obstacles of diverse shapes and the model predictive control concept with the particle swarm optimization algorithm to tackle complex scenarios involving dynamic obstacles [81]. Communication interaction is realized through formation communication, which is contingent upon the formation’s topology. There are two types of topologies in most recent investigations, namely switching topology and fixed topology. However, maintaining a fixed topology for the formation control problem poses a challenge due to factors such as leader failure, constrained communication range, and external interference. Consequently, the topology for the formation control problem is predominantly switched [82]. Yin et al. assert that the information interaction within a flapping-wing cluster is unidirectional, whereby the “follower” receives information from the “leader,” and the “followers” in the rear row receive information from the “followers” in the front row. The implementation of a V-shaped goose formation, as illustrated in Figure 6, involved the alteration of the formation mid-flight to facilitate effective communication and equitable energy distribution during formation flight [83].

**Table 2:** Advantages and disadvantages of the main control methods of formation flight.

Formation Flight Control Method	Advantage	Disadvantage
Leader-follower approach [84,85]	Simple design, easy implementation, and good tracking performance [76]	High dependence on the leader and poor robustness [86]
Based on a behavioral approach [87]	Can adapt to changes in the environment and flexibly adjust the formation to handle multi-target missions	Difficult to model and less stable [86]
Virtual structure approach [78]	High stability	High design and implementation difficulty, high communication quality, strong computing power, and low reliability [88]
Consistent approach [81]	Strong flexibility and robustness for large formations and dense flights [89]	Requires high computational effort and is highly influenced by the environment

In general, formation flight of BFAVs has not received enough attention due to the fact that the technology of flapping-wing vehicles is not as advanced as that of fixed-wing and rotary-wing vehicles. While the control algorithms for formation flight have been extensively researched and applied in the context of rotor-system air vehicles, the utilization of such techniques on BFAV’s physical air vehicles has been comparatively limited. Therefore, further research in this domain is warranted.

There is a close connection and interaction among these three primary controls, namely position control, trajectory tracking control, and formation control. Position control is the basis of trajectory tracking control and formation control, which are critical to the flight stability of BFAV. Trajectory tracking control and formation control need the support of position control to achieve more complex missions. Hence, these three controls are interrelated in BFAV control research and can cooperate for optimal flying performance.

## 4. Summary and Outlook

Bionics as an interdisciplinary discipline has garnered increasing attention in recent years. Within this domain, research on flapping-wing air vehicles (BFAV) has emerged as a hotpot of investigation. While some progress has been made in developing flight control algorithms for BFAVs, achieving high precision and maneuverability in physical air vehicles remains challenging due to the complexity of their models and the multitude of factors that affect control. In light of these findings, this paper presents several perspectives on the future research trajectory for BFAVs:

1. Control of flexible wing deformation. Due to the BFAV’s enormous wingspan, the elastic deformation caused by the flexible wing during flutter will not only affect its aerodynamic performance but also the stability of the system. He et al. created a dynamic model of the flexible wing and applied the Lyapunov direct approach to ensure the stability of the flexible wing system [90]. And the boundary control scheme proposed by Lhachemi et al. ensured the consistent exponential stability of the bending and torsional displacements of the flexible wing [91] and achieved vibration control of the flexible wing. None of these plans, nevertheless, were integrated into the flight control system of the BFAV. Therefore, knowing how to control the flexible wing is an important step in enhancing the BFAV’s aerodynamic performance and stability in order to lessen the impact of flexible wing deformation.
2. Reconfiguration of the controller. The traditional single-control algorithm can hardly meet the flight control requirements of a flapping-wing air vehicle in different flight modes, environmental situations, and missions under a nonlinear, non-constant system. Therefore, the controller of the flapping-wing air vehicle can be modified to extend the applicability and scenarios of BFAV. Aids based on different strategies, such as switching strategy, adaptive strategy, etc., can be used to help the air vehicle choose different controllers or numerous controllers in conjunction with one another to improve the adaptability and scalability of flight control. This is a direction worth exploring.
3. Multi-vehicle synergy and intelligent formation. Flapping-wing air vehicles are not as mature as fixed-wing and rotary-wing air vehicles. Consequently, the accomplishment of high-precision and highly maneuverable missions by a single physical flapping-wing air vehicle is rare, and the instances of such vehicles performing multi-engine synergy and intelligent formation are even scarcer. However, the efficacy and success rate of a single air vehicle are limited when undertaking challenging tasks, such as inspecting vast areas. The integration of intelligent information and multi-vehicle coordination has the potential to enhance the scope of task execution and the reliability of task fulfillment [92]. Consequently, a key research objective for the future is to establish a formation of flapping-wing aerial vehicles that can perform operations efficiently, akin to a flock of birds.

The flight control techniques employed by BFAV are subject to ongoing enhancement to effectively address diverse application scenarios and associated demands in the future. This essay provides three promising avenues for research and exploration: flexible wing deformation control, controller reconfiguration, multi-vehicle collaboration, and intelligent formation.
